# Collective Human Mobility Pattern from Taxi Trips in Urban Area

**DOI:** 10.1371/journal.pone.0034487

**Published:** 2012-04-18

**Authors:** Chengbin Peng, Xiaogang Jin, Ka-Chun Wong, Meixia Shi, Pietro Liò

**Affiliations:** 1 Mathematical and Computer Sciences and Engineering Division, King Abdullah University of Science and Technology, Jeddah, Kingdom of Saudi Arabia; 2 Institute of Artificial Intelligence, College of Computer Science, Zhejiang University, Hangzhou, China; 3 College of Environmental and Resource Sciences, Zhejiang University, Hangzhou, China; 4 Computer Laboratory, Cambridge University, Cambridge, United Kingdom; University of Maribor, Slovenia

## Abstract

We analyze the passengers' traffic pattern for 1.58 million taxi trips of Shanghai, China. By employing the non-negative matrix factorization and optimization methods, we find that, people travel on workdays mainly for three purposes: commuting between home and workplace, traveling from workplace to workplace, and others such as leisure activities. Therefore, traffic flow in one area or between any pair of locations can be approximated by a linear combination of three basis flows, corresponding to the three purposes respectively. We name the coefficients in the linear combination as traffic powers, each of which indicates the strength of each basis flow. The traffic powers on different days are typically different even for the same location, due to the uncertainty of the human motion. Therefore, we provide a probability distribution function for the relative deviation of the traffic power. This distribution function is in terms of a series of functions for normalized binomial distributions. It can be well explained by statistical theories and is verified by empirical data. These findings are applicable in predicting the road traffic, tracing the traffic pattern and diagnosing the traffic related abnormal events. These results can also be used to infer land uses of urban area quite parsimoniously.

## Introduction

Urban traffic has drawn the attention of physicists since more than one decade ago. Generally, there has been two kinds of approaches for the traffic analysis. In microscopic models, some researchers represent vehicles as particles interacting with each other [1], [2], while some others use the cellular automata framework [1], [3], [4]. Based on game theory, the impact of individuals' irregular behaviors on traffic system is also emphasized [5]. On the other hand, from the macroscopic perspective, the idea of fluid dynamics is introduced [1], [6].

In recent years, a new and more fundamental approach for traffic analysis is emerging: human mobility, by drawing statistical inferences from the enormous empirical data [7]–[9]. Several reasons boost the research in this area.

Firstly, the knowledge of the mobility pattern is essential in traffic modeling [10], [11] for simulation, forecasting [12], [13] and control [11]. In addition, by measuring the traffic flow during some time interval to see whether or not it agrees with the verified estimation, the collective mobility analysis can serve as a tool for abnormality definition and detection [14], [15]. Compared to computer vision based detection [16], [17], collective mobility model based abnormality detection can be applied in a much larger scale of area, for example, the whole city.

Secondly, the mobility pattern and the consequential traffic flow can also interact with the land use. The characteristics of traveling strongly influence urban formation, evolving, and future planning [18]–[21], whereas the land use can also affect the urban traffic [22]–[24] and the human mobility [25].

Thirdly, the better understanding of human mobility can help to more easily control the spreading of contagious diseases by limiting the contact among individuals [26], since the transmission of infected people from one place to another is an important way to infect the susceptible ones, either in a small scale area [27],[28] or from a worldwide viewpoint [29]–[31]. Similar theories hold for viruses contamination with malicious code among wireless communication devices [32], [33].

Due to the high importance of human mobility research, and the availability of the large amount of empirical data as a consequence of the prevalence of wireless communication devices, researchers become more and more interested in the statistical features of human mobility pattern via real world data [34]. Ref. [7] and Ref. [9] suggest that human travels are reminiscent of Lévy Flights [35] according to the trajectories of bank notes and taxies respectively, while Ref. [36] reports some variances by the GPS information from volunteers. These differences are later recognized as a result of the periodic pattern of individual's traveling [8] and recently Ref. [37] discovers up to 93% of total time when individual locations are predictable in their data set, which contains trajectories of mobile phone users. For taxi trips, Ref. [38] studies the distribution of the travel distances and time.

Nevertheless, previous statistical inferences of human mobility mostly focus on individual level, while this article analyzes the citizens' collective dynamics in the urban area. In our research, based on the traveling purposes, we discovered three distinct basis patterns for collective traffic flow regardless of the location. In addition, a distribution is revealed that can characterize the fluctuation of the traffic flow at any time in each location. As mentioned above, these findings can be useful for urban planning, traffic estimation and anomalous detection. Further studies on interaction between different areas will provide a more detailed collective mobility model, and would additionally benefit the research on epidemic spreading in urban area.

## Analysis

### Data Description and Background Assumptions

In this research, the data [39] are collected from about two thousand taxies operating within the urban area of Shanghai, China. These data mainly focus on the central part of city, and the population in this part is about seven million according to the fifth national population census [40]. The information about when and where passengers were picked up and dropped off can be retrieved from the raw data, and every pair of picking and dropping information is defined as a taxi trip. The data set includes about 1.58 million taxi trips. The longitude and latitude location information in the data by GPS is converted to positions in a planar coordinate system, with the city landmark Oriental Pearl Tower as the origin. For the ease of analyzing and representing, the urban area is divided into squares, similar to a chessboard. The side lengths of each square is identically 200 meters. In our context, each location corresponds to one of these squares. More details can be found in Appendix S1.

### Basis Traffic Flows: the Constancy

As we know, even a 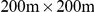 area in a city can possess land of several different types, for example, containing schools, shops and apartments at the same time. In this section, we will discuss how to categorize the taxi trips according to the traveling purposes, and then use these categories to infer the land use composition for each square.

First of all, we consider the taxi trip categorization. People setting out in the same location would possibly have different purposes: some may go to workplaces while some others may go for entertainment. Meanwhile, for trips belonging to the same category but in different locations, the collective pattern should be similar, regarding to the departure and arrival time in a large amount of data. For example, if the number of trips between residential area and workplaces (for commuting purpose) reaches the highest at 8:00 am (going to work) and 5:00 pm (getting off work), then the number of trips in this category in any place would peak almost at the same time, although the scale may be different.

In short, we can define a set of basis collective patterns, each of which corresponding to a trip category respectively. Then linear combinations of these patterns can describe the macro traveling pattern of each location. Finally, the coefficients in a linear combination can reflect the land uses of the location.

Directly from the taxi data, we can only calculate the macro patterns. Therefore, we should adopt appropriate inference methods to find the basis patterns and the coefficients for each location.

To represent our method more formally, we define 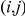 to index the square in 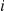
th row and 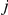
th column among all the squares divided within the city. If 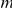 is the number of rows and 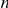 is the number of columns for squares in the map, then 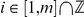, and 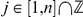. Let 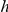 be the number of time slots, normally 24 for one day. Therefore for location 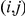, the numbers of departure and arrival trips (macro pattern) along time each day can be represented by a 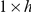 vector 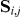, which is easy to calculate. We can also define a set of 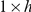 vectors containing normalized numbers of trips along time: 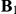, 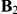, 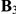, 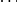, 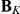, each for one basis pattern that we seek for.

The macro pattern is a linear combination of basis patterns, so we have
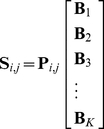
(1)where 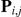 is a row vector containing 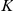 coefficients for the linear combination on the right-hand side.

By taking all the locations into account, it can also be written as
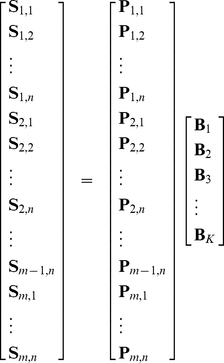
(2)and abbreviated as



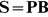
(3)Because the two matrices on the right-hand side of Eq. (3) are unknown, there are many matrix decomposition methods that may apply. However, according to the physical meaning of 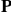 and 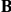, all the entries of these two matrices should be nonnegative. Therefore, we choose nonnegative matrix factorization (NMF) [41], [42] for the decomposition.

In our context, it is a method to factorize a matrix 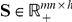 into two nonnegative factors 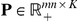 and 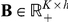 approximately. By this approach, we can find the basis patterns (the row vectors of 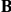) and the parameter vectors (the row vectors of 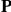) simultaneously. As vector 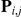 (the 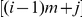
th row of matrix 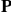) is only responsible for vector 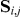 (the 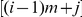
th row of matrix 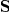), in fact, each element of 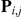 denotes the scale of traffic flow with respect to the corresponding category, in location 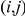. Hence, we also call these elements the traffic power because they reflect how strong the traffic flows of different categories are.

Now the only thing left is to determine 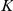, the number of the basis patterns.

**Figure 1:**
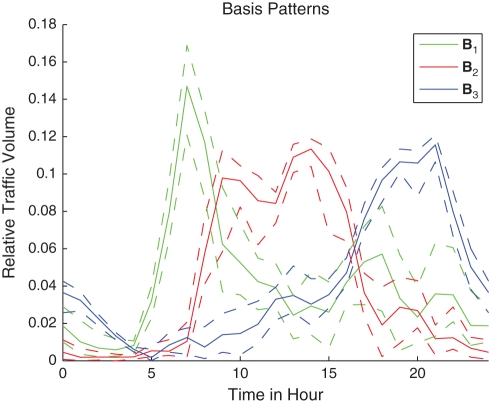
Basis Pattern B: Green is B1, Red is B2, and Blue is B3. Solid Lines Represent the Mean 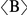, while Dashed Lines Represent the Positive and Negative Deviations Averaged on Different Days.

From the algorithmic perspective, we noticed that NMF starts with random initial conditions [41]. By experiments on the taxi data with many different random initial conditions, we find that only when 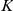 equals 3, the factorization results can be stable. This fact indicates that with parameter 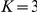, NMF can find out statistically significant characteristics for the data, and Fig. 1 demonstrates the resulted basis pattern 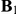, 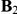 and 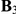.

On the other hand, from the land-use and trip-category perspective, 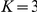 is a reasonable choice in categorizing trip purposes.

There are several land-use definitions related to the topic of mobility. For example, each place may be classified as a residential (home), working, shopping, or recreational location [27]. It may also be regarded as one of the following types: a residential area, a workplace, a commercial zone, a recreation area and educational facilities [43]. In Ref. [44], these types are simplified into workplace, home and shop. Specifically for the city of Shanghai based on GIS information, Ref. [45] refers to the land types including residence, industry, agriculture, roads, water, land for construction and other urban land. In our context, we can simplify the land-use definition to be: residences, workplaces and others. Here workplaces shall include any industrial and office workplaces as well as schools, and other places can include shopping and recreational facilities, hospitals, etc.

For trips, some scientists categorize these individual activities into several orientations: family, work, leisure and service-based movement [46]. Similarly, according to our land-use definition, we can use three purpose-based categories for the trips: commuting between home and workplace (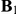), business traveling between two workplaces (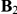), and trips from or to other places (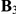). This representation is in accordance with the algorithmic result in Fig. 1. Take a typical workday as an example, based on our three categories, the major traffic flows in the city are supposed to be as follows: those from home to workplaces in the early morning (green line), from one workplace to another in the daytime (red line), from workplace to home or to other places at dusk (green line again), and those between other places and home in the night (blue line).

Therefore, 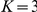 is an effective and reasonable choice.

In the following sections with 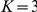, for clarity, we will use 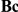, 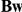 and 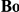 to replace 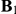, 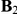 and 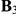 respectively:
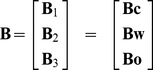
(4)


We also use 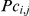, 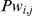 and 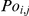 to represent the three entries in vector 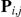:
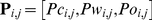
(5)


Appendix S2 describes the detailed implementation about applying NMF to this problem. The basis pattern on different days are averaged to 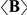. Then, 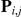, the traffic power, can be recalculated based on 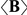 for different day. If it variants in an acceptable interval day by day, the daily average of 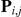, represented by 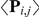, can indicate the land use of location 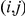. For example, if 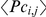 is large, then the traffic flow corresponding to basis pattern 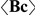 is large, suggesting that location 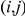 serves mainly for residences or workplaces, while if 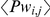 is the largest, we can be quite sure that this location is mainly for workplaces. In addition, if the variation of 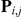 on some day goes out of the acceptable interval, it indicates that something abnormal happens on that day. This feature can be helpful for anomaly detection on human activities in a large area. In the next section, we will analyze the variance of 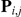, to determine what is an acceptable interval.

### Daily Traffic Power: the Variation

Typically in a city, the volume of the traffic flow is quite regular everyday [8]. However even for the same time in the same location but on different days, the volume is vulnerable to change within a certain range. This section is devoted to analyze how 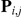 fluctuates everyday. In this case, 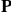 is calculated from the average basis pattern 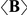 according to Appendix S2.

We define a random variable 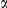 to represent the relative variance of the traffic power.

The empirical distribution function of 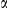 can be simply extracted from a collection of the following expressions in different locations on different days:
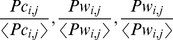
(6)where 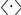 means the daily average, as we have used.

We also find the theoretical distribution function of 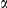, which is more complex.

First, we try to find 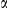 only for the first category of trips in location 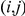. We define 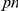 as the potential population that may affect the first-category traffic in this location, and 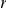 as the probability (ratio) that an individual in the population finally becomes part of that traffic flow. Then the number of such trips follows a binomial distribution:
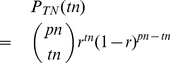
(7)where 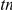 can be any non-negative integer less than 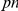. Because it is a binomial distribution, the corresponding CDF can be written in terms of the beta functions:
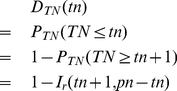
(8)where 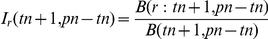. 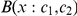 is the incomplete beta function as 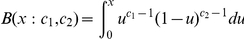 and 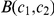 is the beta function as 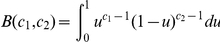. Eq. (8) is strictly equal when 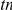 is a positive integer, while for a real positive number of 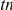, we may use this approximation:



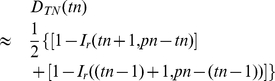
(9)According to the definition, 
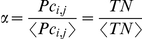
, where 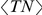 is equivalent to 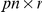 by the property of expectation of the binomial distribution, and can be treated as a constant for a given location. Therefore, the probability density function (PDF) of 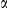 is:
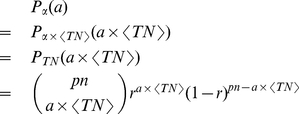
(10)where 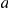 should satisfy the condition that 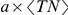 is a non-negative integer. The cumulative distribution function (CDF) is



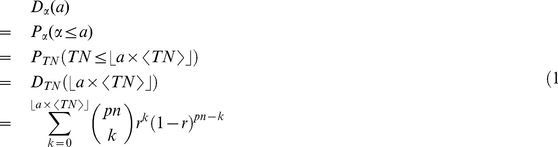
(11)where 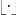 where represents the floor function. We call this distribution the normalized binomial distribution of 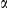. As listed in Appendix S3, the moment generation functions of 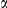 indicate that 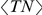 plays an essential role in the distribution. Numerical simulations also provide evidence that the distribution of 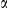 is strongly affected by 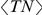 (the product of 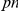 and 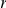), but is almost irrelevant to 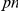 or 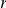 alone. Therefore, we can assign an constant integer 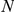 to 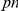.

Let 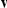 be a vector containing all the possible values of 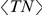. Then the PDF of 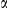 with 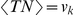 can be written in this form
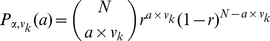
(12)and the CDF is



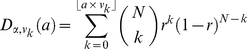
(13)


Finally, we discuss how to make 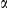 representative for variations of any traffic category in any location. We define a vector 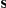, in which each entry 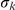 denotes the proportion of traffic flow corresponding to 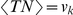. Then for a randomly selected traffic flow, when the average number of trips 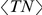 is not given, a general expression for the CDF of 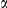 is
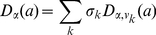
(14)


By beta approximation as in Eq. (9), it can be written into a continuous version
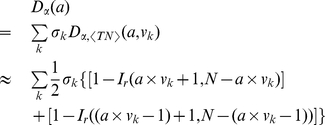
(15)


## Results

In this section, we demonstrate how our theoretical results are supported by the empirical investigation.

**Figure 2:**
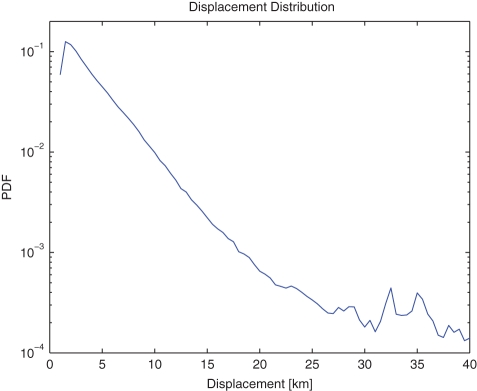
Traveling Distance Distribution.

**Figure 3:**
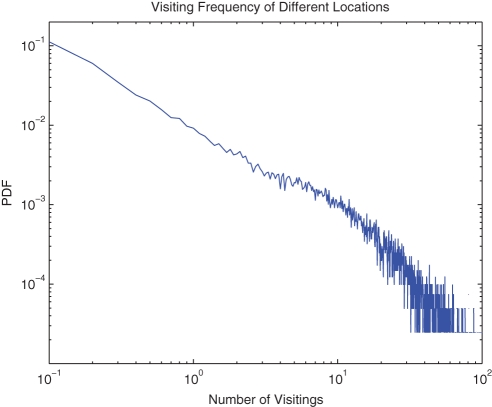
Visiting Frequency Distribution of Different Locations.

**Figure 4:**
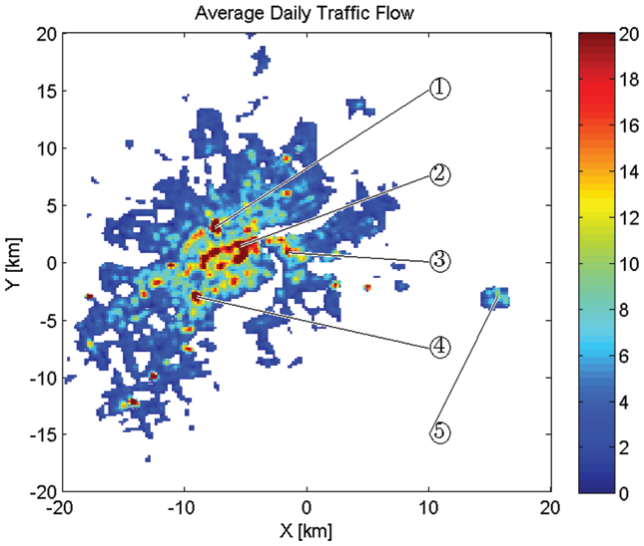
The Average Traffic Flow of Each Location, and the Tags Corresponding to Following Locations: ◯1 Shanghai Railway Station; ◯2 Nanjing Road & People's Square; ◯3 Lujiazui Finance & Trade Zone; ◯4 Shanghai South Railway Station; ◯5 Pudong International Airport.

The general characteristics of our data set, such as the displacement distribution in Fig. 2 and the visiting frequency distribution in Fig. 3, are similar to others' [8], [38]. The plot of daily traffic flow in Fig. 4 exhibits some hot areas by red, including the most flourishing commercial street Nanjing Road as the largest red block, Shanghai Railway Station, Shanghai South Railway Station, Lujiazui Finance & Trade Zone, etc. The largest isolated area in blue is the Pudong International Airport.

Without any intentional intervention, by NMF with random initial values, we find that the normalized basis pattern on workdays is generally quite similar (Fig. 1). Therefore, we can use the traffic power 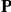 to analyze the mean and the deviation of daily traffic.

**Figure 5:**
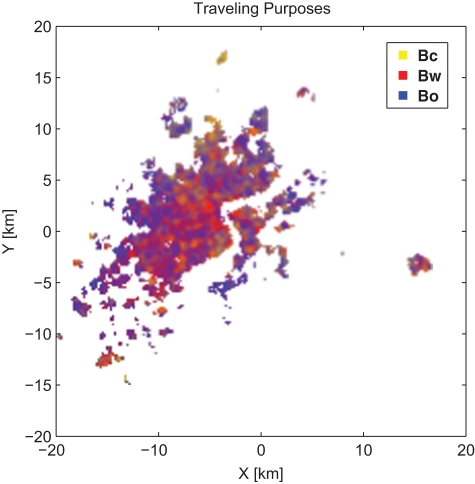
The Average Component Proportions of 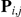 in Each Location, Equivalent to the Categorical Proportion of the Traffic.

**Figure 6:**
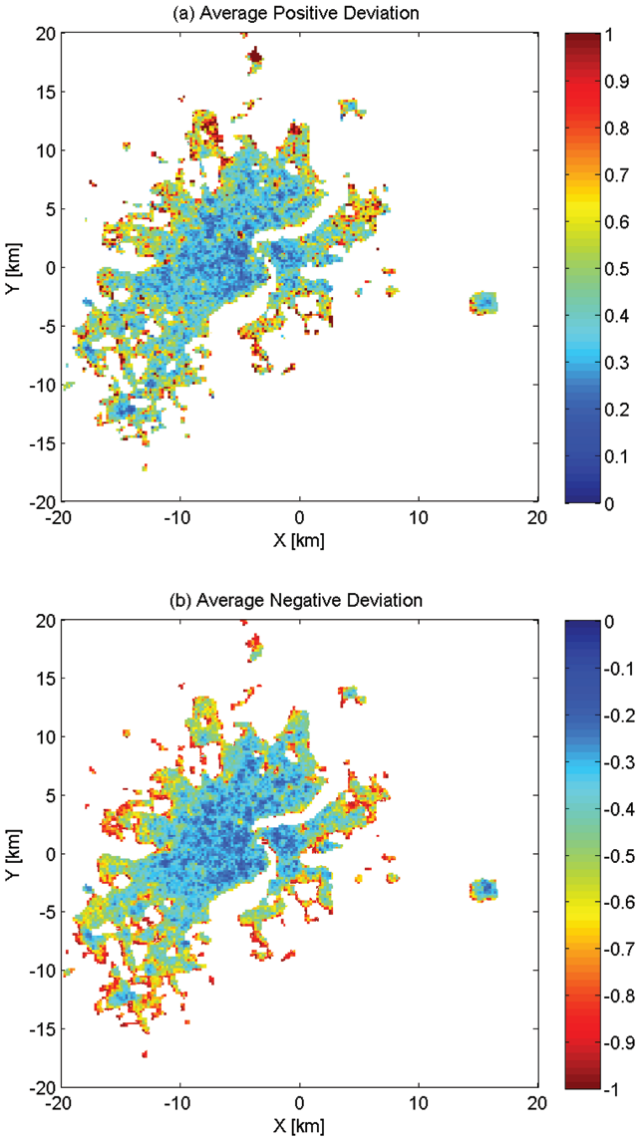
The Relative Deviation for Components of 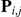 in Each Location: (a) the Average Positive Deviation; (b) The Average Negative Deviation.

**Figure 7:**
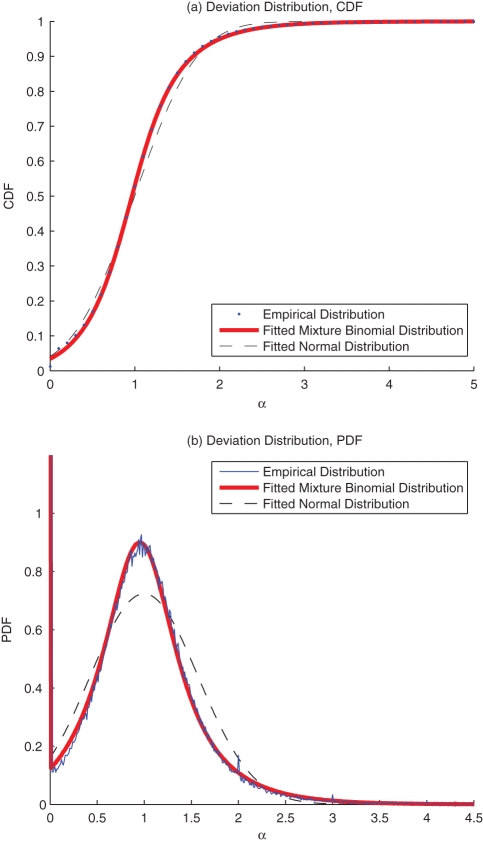
The Distribution of the Relative Deviation for Components of 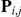: (a) CDF; (b) PDF.

**Figure 8:**
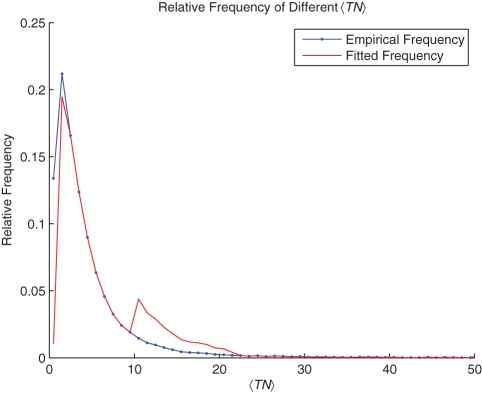
The Parameters for the Distribution.

**Figure 9:**
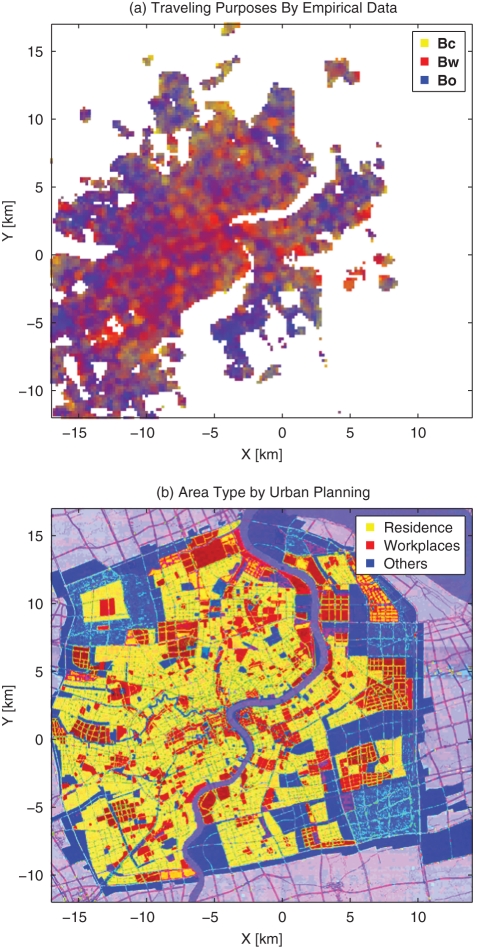
Comparing the Empirical Data to Urban Planning Map: (a) the Area Type from Urban Planning [**47**] for Central Part of the City; (b) the Average Categorical Proportion of Traffic for Central Part of the City.

In Fig. 5, the three components of 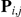 in every location is normalized and represented by yellow, red and blue respectively. For example, a location in yellow color means the traffic flow of the first category (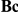: commuting between home and workplace) is dominant there. Mixed colors in some places indicate a mixture of traffic flows of different categories. It is noticeable that in area where the traffic flow is large, the positive (Fig. 6(a)) and negative (Fig. 6(b)) deviation of the traffic power 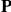 is quite small. The distribution of this deviation can be represented accordingly by Fig. 7(a) and Fig. 7(b), which is fitted well with Eq. (15). This fitting result is quite different from the best fitted normal distribution by the central limit theory, which verifies Eq. (14) and Eq. (15) that 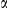 should be a collection of random variables following a set of distributions with different parameters. The proportion of traffic flow with 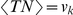 is 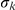, as plotted in Fig. 8. Here we limit each 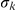 to be no larger than twice of the empirical value. According to the result in Fig. 7, for the whole city, 80% of the deviations are within the range of 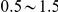. Although the lengths of vectors 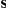 and 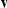 are identically 50 in our estimation, the number of active pairs (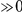) of 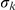 and 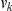 is only about 10, and this number can be reduced if we only calculate for a small area given the sufficient amount of data. In short, we can see that Eq. (15) can be a reasonable approximation for the relative deviation of the daily traffic flow. Fig. 9(b) presents the components of 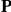 for the central part of the city in comparison with the urban planning map for Year 2004–2020 in Fig. 9(a). Generally, it can be seen that the residence area have a large volume of traffic with respect to 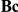 and 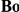, corresponding to trips between home and workplaces and trips for other purposes, while in the workplace area especially for business, there are lots of flows corresponding to the second category 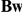, and in the remaining area, the third one 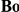 is quite significant. We should note that the urban planning map (2004–2020) is not an exact description for the land uses of Year 2007, and consequently, the patterns of the two figures may not agree well in some small areas. For example, the red patch around point 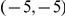 in Fig. 9(a) is planned as an industrial land, namely, workplace in our context, while in fact it was a construction site for Expo 2010 Shanghai China with very few taxi traffic in Year 2007. Yet it is still reasonable for a construction site to have the major taxi flows of type 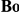 as shown in Fig. 9(b) because in the evening workers would be very likely to go out for recreation, entertainments, etc.

In addition, we can see how the government planning [47] is affected by what it is now. For example, Nanjing Road and near by is the largest block with high traffic throughput, and traffic flows are constituted mainly by those of workplaces related (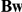) and other facilities related (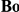) categories. In the planning, it is designed to be a public activity center for administrative, business and shopping purposes. Lujiazui is another similar but smaller zone, which is planned mainly for business and shopping centers.

## Discussion

In this research, we find that the traffic on workdays can be divided into three categories according to the different purposes: commuting between home and workplaces, traveling from workplace to workplace, and others such as leisure activities. Each of these categories has a highly distinguishable basis pattern: 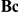, 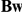 or 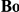. The relative daily deviation of the traffic flow in each category can be modeled as Eq. (14), which is a mixture of normalized binomial distributions, with a continuous approximation as Eq. (15).

This basis pattern theory is applicable to data sets containing the beginning and ending information of trips, such as the bicycle departure and arrival data [48], cell phone based mobility information [8], GPS based data, etc.

The first contribution of this research is, it provides a very economical approach to understand how the urban traffic at different locations are composed from the three categories. For instance, a large 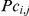 means there is a large portion of traffic between home and workplaces at location 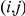. This theory can also help to infer the land use composition by a quite easy, real-time, and automated way. For example, the evidence of a large 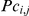 everyday indicates location 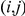 is mainly for residential or working purpose, while a large 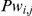 can imply that it has lots of workplaces. A mixture of different land uses in a single location can be found by this method as well.

Second, based on the NMF approach, the time series of the total traffic at any location can be expressed as a linear combination of the basis patterns. Therefore, we can compress the traffic data of a large area into a very small data size, but still with a quite high resolution. Namely, we only need to store the global basis patterns, and for each location, we use a small vector for the traffic power to represent how strong each basis pattern is.

Third, we find that the distribution of the relative deviation is not a normal distribution, indicating that the random variable 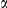 is not identical from one place to another, or from time to time. The significance of Eq. (14) and Eq. (15) is, they provide an expression of how traffic fluctuates for various unknown positions and time intervals. This description of relative deviation can also be helpful to estimate the change of the traffic flow, which would be important in traffic predicting, controlling and urban planning.

Finally, with the deviation distribution, we can not only predict the change of traffic, but also diagnose the abnormality of the traffic: where, when, why, and how. The first two functions are obvious, while ‘why’ abnormal can be disclosed by the traffic power, and ‘how’ abnormal can be revealed by the probability of the deviation. For example, if some traffic flow is very abnormal one day, the probability density of the variance on that day should be very small.

Our analysis focusing on the traffic flows in different locations on different workdays. Our results can also be extend to the traffic on a road. The road traffic is a summation of the traffic passing this road from several sources and to several destinations. Therefore, the volume and the deviation of the road traffic flow can also be explained in our framework.

## Supporting Information

Appendix S1 More on Data Description and Background Assumptions.
(PDF)

Appendix S2 Implementation Details about the Factorization.
(PDF)

Appendix S3Moment Generation Function of 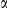.(PDF)
