# Correction: Collective Human Mobility Pattern from Taxi Trips in Urban Area

**DOI:** 10.1371/annotation/f0d48839-ed4b-4cb2-822a-d449a6b4fa5d

**Published:** 2012-08-17

**Authors:** Chengbin Peng, Xiaogang Jin, Ka-Chun Wong, Meixia Shi, Pietro Liò

There is an error in author Ka-Chun Wong's affiliations. The author should be affiliated with:

5. Department of Computer Science, University of Toronto, Toronto, Ontario, Canada,

6. Terrence Donnelly Centre for Cellular and Biomolecular Research, University of Toronto, Toronto, Ontario, Canada,

