# Supplementary material for: Collective Human Mobility Pattern from Taxi Trips in Urban Area
Source: PLoS One. 2012 Apr 18;7(4):e34487. doi: 10.1371/journal.pone.0034487 (PMC3329492; doi:10.1371/journal.pone.0034487)
Supplement: Appendix S1 — More on Data Description and Background Assumptions. (PDF) [file pone.0034487.s001.pdf]

Collective Human Mobility Pattern from Taxi Trips in Urban Area

Chengbin Peng[1,2], Xiaogang Jin[2], Ka-Chun Wong[1], Meixia Shi[3], Pietro Liò[4,*]
1 Mathematical and Computer Sciences and Engineering Division, King Abdullah University of Science and Technology, Jeddah, Kingdom of Saudi Arabia
2 Institute of Artificial Intelligence, College of Computer Science, Zhejiang University, Hangzhou, China
3 College of Environmental and Resource Sciences, Zhejiang University, Hangzhou, China
4 Computer Laboratory, Cambridge University, Cambridge, United Kingdom
∗ **E-mail: Pietro.Lio@cl.cam.ac.uk**

Supporting Information

S1 More on Data Description and Background Assumptions

When dividing the urban map into locations (squares), if the side length of the square is large, the outcome resolution is low. However, if the the side length is small, a single building for example a mall, may be split into several neighboring locations, and the data intensity of each location is reduced, so that the time series tend to be incomplete. In addition, when a position of the trips is assigned naively to locations, the position information is likely to be distorted. For example, if an ending position of a trip is at the corner of a square location, and is naively assigned, it roughly excludes the possibility that the real destination of the passenger may be in one of the other three neighboring locations.

To solve these problems, rather than simply calculate the number of departure and arrival trips for each location, the concept of a passenger's real source and destination points is introduced. We assume each of them, either source or destination point, is an uniformly distributed random variable falling within a circle centered at the recorded trip position. The radius of the circle is 300 meters. This distance is about 5-minute walk, and is typically the maximum distance one would like to walk before or after the taxi trip. Then the number of trips in a location is calculated by summing the cumulative probability within the location for all the points.

For example, in Fig. 1, for a given recorded taxi position (RTP), the real source or destination point can probably be any point inside the blue circle. Therefore, the contribution of this RTP to the traffic of location (1,2) is defined as the ratio of the red area to the whole area of the blue circle. Similarly, its contribution to location (3,2) is the ratio of the purple area to the area of the circle.

Note the choice of the radius is not sensitive. As we choose a radius of 200 meters, the basis traffic flows and the daily fluctuations are similar to the result presented in this paper.

References

Figure Legends

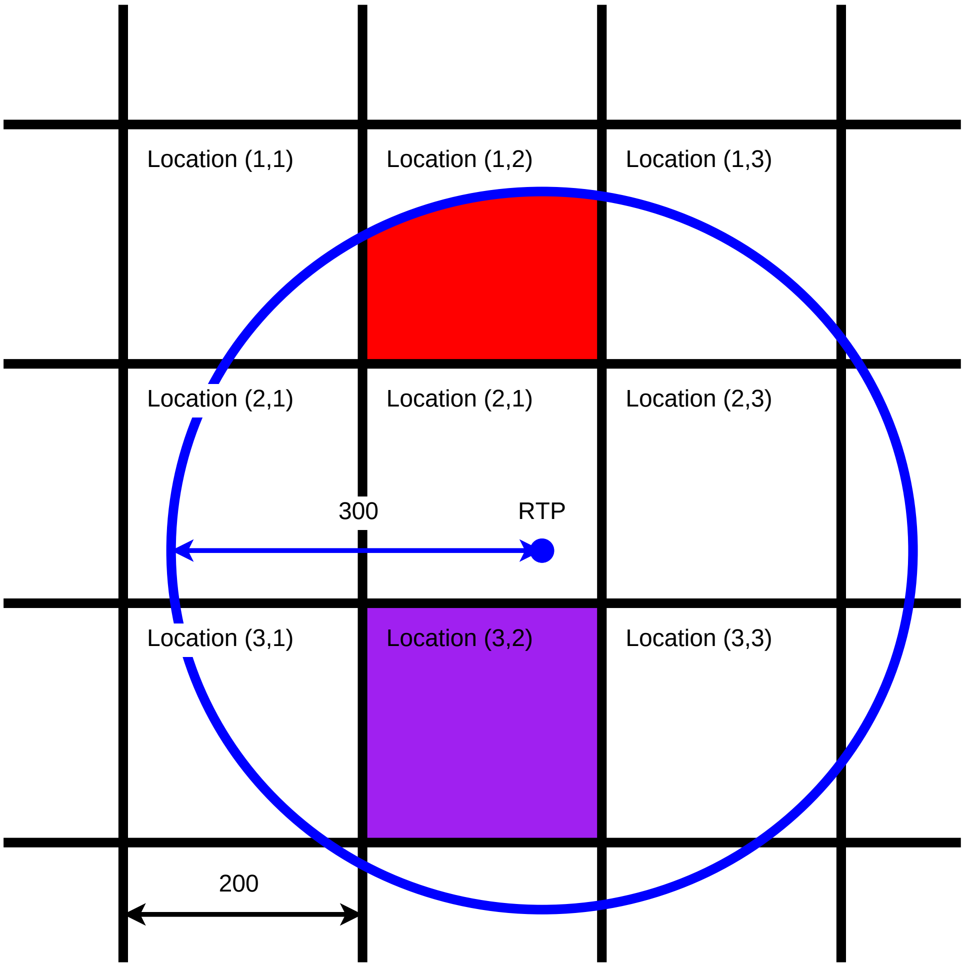

Figure 1. Example of the Relationship between Recorded Taxi Position (RTP) and the Passenger's Real Source or Destination Point. Unit: meter.
