# Supplementary material for: Collective Human Mobility Pattern from Taxi Trips in Urban Area
Source: PLoS One. 2012 Apr 18;7(4):e34487. doi: 10.1371/journal.pone.0034487 (PMC3329492; doi:10.1371/journal.pone.0034487)
Supplement: Appendix S2 — Implementation Details about the Factorization. (PDF) [file pone.0034487.s002.pdf]

Collective Human Mobility Pattern from Taxi Trips in Urban Area

Chengbin Peng[1,2], Xiaogang Jin[2], Ka-Chun Wong[1], Meixia Shi[3], Pietro Liò[4,*]
1 Mathematical and Computer Sciences and Engineering Division, King Abdullah University of Science and Technology, Jeddah, Kingdom of Saudi Arabia
2 Institute of Artificial Intelligence, College of Computer Science, Zhejiang University, Hangzhou, China
3 College of Environmental and Resource Sciences, Zhejiang University, Hangzhou, China
4 Computer Laboratory, Cambridge University, Cambridge, United Kingdom
∗ E-mail: Pietro.Lio@cl.cam.ac.uk

Supporting Information

S2 Implementation Details about the Factorization

S2.1 Normalize Daily Basis Patterns

The basis patterns from different workdays extracted by NMF are usually very similar, because of the regularity in our life [1]. However, they are not identical. To obtain the most accurate daily-averaged basis pattern $\langle \mathbf{B} \rangle$, we should minimize the scale differences by normalization. The normalization on a pattern $\mathbf{B}$ by multiplying some coefficient $\mathbf{x}$ does not affect the accuracy of NMF, if $\mathbf{P}$ is adjusted correspondingly according to Eq. 1.

$$\begin{aligned} \mathbf{S} =& \mathbf{P} \begin{bmatrix} \mathbf{Bc} \\ \mathbf{Bw} \\ \mathbf{Bo} \end{bmatrix} \\ =& \begin{bmatrix} Pc, Pw, Po \end{bmatrix} \begin{bmatrix} \mathbf{Bc} \\ \mathbf{Bw} \\ \mathbf{Bo} \end{bmatrix} \\ =& \begin{bmatrix} \frac{1}{xc}Pc, \frac{1}{xw}Pw, \frac{1}{xo}Po \end{bmatrix} \begin{bmatrix} xc\mathbf{Bc} \\ xw\mathbf{Bw} \\ xo\mathbf{Bo} \end{bmatrix} \end{aligned} \tag{1}$$

In the following part, by taking $\mathbf{Bc}$ as an example, we demonstrate how to find the corresponding scaling factor $\mathbf{xc}$ for the normalization of $\mathbf{Bc}$. xc_d is an entry of $\mathbf{xc}$ with respect to the dth day, and $Bc_{d,t}$

is the basis traffic pattern between residence and workplaces at the tth hour of dth day.

$$\begin{aligned}
\min\ f(\mathbf{xc}) &= \sigma_d(xc_d \times Bc_{d,t}) \\
&= \frac{1}{nd}\sum_{d=1}^{nd}\sum_{t=1}^{h}[xc_d \times Bc_{d,t} \\
&\qquad - \langle xc_{d'} \times Bc_{d',t}\rangle_{d'}]^2 \\
&= \frac{1}{nd}\sum_{d=1}^{nd}\sum_{t=1}^{h}[xc_d \times Bc_{d,t} \\
&\qquad - \frac{1}{nd}\sum_{d'=1}^{nd}(xc_{d'} \times Bc_{d',t})]^2 \\
\text{s.t. } & xc_{d'} > 0,\ d' \in [1, nd] \cap \mathbb{Z} \\
& \sum_{d'=1}^{nd}(xc_{d'} \times \sum_{t=1}^{h} Bc_{d',t}) = 1
\end{aligned} \tag{2}$$

where $\sigma_d(\cdot)$ and $\langle\cdot\rangle_d$ stands for the variance function and the average function respectively along the subscript d. nd means the number of days taken into account and is also the length of vector $\mathbf{xc}$, and h is the number of time slots in one day, which typically equals 24. The two constrains are necessary to avoid $xc_{d'}$ to be non-positive or infinitely close to 0.

By Eq. (2) and two similarly approaches, we can find the optimized scaling coefficients $\mathbf{xc}$, $\mathbf{xw}$ and $\mathbf{xo}$ [2]. We can use these coefficients to normalize daily basis pattern correspondingly not only for averaging, but also for identifying outliers. This is because for outliers, even after normalization, their deviation from $\langle\mathbf{B}\rangle$ are still large compared to others.

Finally, we can also apply an element-wise division on each column of $\langle\mathbf{B}\rangle$ by the row sum of $\langle\mathbf{B}\rangle$.

$$\langle\mathbf{B}\rangle \leftarrow \begin{bmatrix} \langle\mathbf{Bc}\rangle./\sum_{t=1}^{h}\langle Bc_t\rangle \\ \langle\mathbf{Bw}\rangle./\sum_{t=1}^{h}\langle Bw_t\rangle \\ \langle\mathbf{Bo}\rangle./\sum_{t=1}^{h}\langle Bo_t\rangle \end{bmatrix} \tag{3}$$

Then, the row sum of the resulted $\langle\mathbf{B}\rangle$ is 1.

S2.2 Factorization

Since $K = 3$ is an appropriate choice, $\mathbf{S}_{i,j}$ can be written as a linear combination of three components: $\mathbf{Bc}$ for commuting between home and workplace, $\mathbf{Bw}$ for business travel between two workplaces, and $\mathbf{Bo}$ for other trips.

Firstly, we apply NMF [3, 4] for daily data, so that we have a basis pattern per day. Secondly, we normalize the daily basis pattern (Appendix) and exclude the outliers. Thirdly, we average the remaining basis patterns to $\langle\mathbf{B}\rangle$, and use $\langle\mathbf{B}\rangle$ with $\mathbf{S}_{i,j}$ to determine the traffic power $\mathbf{P}_{i,j}$ by minimizing the L1-norm of the error vector $\mathbf{S}_{i,j} - \mathbf{P}_{i,j} \times \langle\mathbf{B}\rangle$.

$$\begin{aligned}
\min\ & \sum_{t=1}^{h} |\{\mathbf{S}_{i,j} - \mathbf{P}_{i,j} \times \langle\mathbf{B}\rangle\}_t| \\
\text{s.t. } & Pc_{i,j} \geq 0; \\
& Pw_{i,j} \geq 0; \\
& Po_{i,j} \geq 0;
\end{aligned} \tag{4}$$

where the notation $\{\cdot\}_t$ indicates the tth element of the vector in the brackets. If we define scalar variables $q_t \geq |\{\mathbf{S}_{i,j} - \mathbf{P}_{i,j} \times \langle \mathbf{B} \rangle\}_t|, t \in [1, m] \cap \mathbb{Z}$, then this problem can be formulated into linear programming for higher efficiency.

$$\begin{aligned} \min\ & \sum_{t=1}^{h} q_t \\ \text{s.t.}\ & Pc_{i,j} \geq 0; \\ & Pw_{i,j} \geq 0; \\ & Po_{i,j} \geq 0; \\ & |\{\mathbf{S}_{i,j} - \mathbf{P}_{i,j} \times \langle \mathbf{B} \rangle\}_t| \leq q_t, t \in [1, m] \cap \mathbb{Z} \\ & - q_t \leq |\{\mathbf{S}_{i,j} - \mathbf{P}_{i,j} \times \langle \mathbf{B} \rangle\}_t|, t \in [1, m] \cap \mathbb{Z} \end{aligned} \tag{5}$$

References

1. González M, Hidalgo C, Barabási A (2008) Understanding individual human mobility patterns. Nature 453: 779–782.

2. Nocedal J, Wright S (1999) Numerical optimization. Springer.

3. Lee D, Seung H (1999) Learning the parts of objects by non-negative matrix factorization. Nature 401: 788–791.

4. Lin C (2007) Projected gradient methods for nonnegative matrix factorization. Neural computation 19: 2756–2779.
