# Supplementary material for: Collective Human Mobility Pattern from Taxi Trips in Urban Area
Source: PLoS One. 2012 Apr 18;7(4):e34487. doi: 10.1371/journal.pone.0034487 (PMC3329492; doi:10.1371/journal.pone.0034487)
Supplement: Appendix S3 — Moment Generation Function of . (PDF) [file pone.0034487.s003.pdf]

Collective Human Mobility Pattern from Taxi Trips in Urban Area

Chengbin Peng[1,2], Xiaogang Jin[2], Ka-Chun Wong[1], Meixia Shi[3], Pietro Liò[4,*]
1 Mathematical and Computer Sciences and Engineering Division, King Abdullah University of Science and Technology, Jeddah, Kingdom of Saudi Arabia
2 Institute of Artificial Intelligence, College of Computer Science, Zhejiang University, Hangzhou, China
3 College of Environmental and Resource Sciences, Zhejiang University, Hangzhou, China
4 Computer Laboratory, Cambridge University, Cambridge, United Kingdom
∗ E-mail: Pietro.Lio@cl.cam.ac.uk

Supporting Information

S3 Moment Generation Function of α

In this section we discuss α as a random variable following a normalized binomial distribution described by Eq. (10) and Eq. (11). Then the moment generating function of α is

$$\begin{aligned}
&M(g)\\
=&\langle e^{g\times\alpha}\rangle\\
=&\sum_{\alpha\langle TN\rangle=0}^{pn} e^{g\times\alpha}\binom{pn}{\alpha\langle TN\rangle} r^{\alpha\langle TN\rangle}(1-r)^{pn-\alpha\langle TN\rangle}\\
=&\sum_{\alpha\langle TN\rangle=0}^{pn} \binom{pn}{\alpha\langle TN\rangle}(r\times e^{\frac{g}{\langle TN\rangle}})^{\alpha\langle TN\rangle}(1-r)^{pn-\alpha\langle TN\rangle}\\
=&(r\times e^{\frac{g}{\langle TN\rangle}}+(1-r))^{pn}\\
=&(r\times e^{\frac{g}{pn\times r}}+(1-r))^{pn}
\end{aligned} \tag{1}$$

and consequently, the first, second and third moments are as follows.

$$\begin{aligned}
&\mu_1\\
=&M'(g)|_{g=0}\\
=&(r+(1-r))^{pn-1}\\
=&1
\end{aligned} \tag{2}$$

$$\begin{aligned}
&\mu_2\\
=&M''(g)|_{g=0}\\
=&(pn-1)\frac{1}{pn}+\frac{1}{\langle TN\rangle}\\
\approx&\frac{1}{\langle TN\rangle}
\end{aligned} \tag{3}$$

$$
\begin{aligned}
&\mu_3\\
=&M'''(g)|_{g=0}\\
=&\frac{pn-1}{pn}[(pn-2)r\frac{1}{\langle TN\rangle}+\frac{2}{\langle TN\rangle}]\\
&+\frac{1}{\langle TN\rangle}[(pn-1)r\frac{1}{\langle TN\rangle}+\frac{1}{\langle TN\rangle}]\\
\approx&1+\frac{2}{\langle TN\rangle}+\frac{1}{\langle TN\rangle}(1+\frac{1}{\langle TN\rangle})\\
=&1+\frac{3}{\langle TN\rangle}+\frac{1}{(\langle TN\rangle)^2}
\end{aligned} \tag{4}
$$

Typically pn is quite large, so that $pn \approx pn-1 \approx pn-2$, and the approximations above are valid.

References
